# Phase I study of the combination of losoxantrone and cyclophosphamide in patients with refractory solid tumours

**DOI:** 10.1038/sj.bjc.6600123

**Published:** 2002-02-12

**Authors:** B C Goh, E E Vokes, A Joshi, M J Ratain

**Affiliations:** Department of Medicine, Section of Hematology/Oncology, University of Chicago, 5841 S. Maryland Avenue, Illinois, IL 60637, USA; Cancer Research Center, University of Chicago, 5841 S. Maryland Avenue, Illinois, IL 60637, USA; Committee on Clinical Pharmacology, University of Chicago, 5841 S. Maryland Avenue, Illinois, IL 60637, USA; Clinical and Experimental Pharmacology, Genentech Inc., 532 Forbes Blvd, San Francisco, California, CA 94080, USA

**Keywords:** combination, losoxantrone, cyclophosphamide, cardiotoxicity

## Abstract

Losoxantrone is a DNA intercalator that was developed with the potential to replace anthracyclines. The recommended single agent dose of losoxantrone is 50 mg m^−2^ every 3 weeks. We conducted a phase I study of losoxantrone and a fixed dose of cyclophosphamide on a q3 weekly schedule. Forty-nine patients were enrolled, of which 46 were evaluable for toxicity. The dose-limiting toxicity was neutropenia at the maximum tolerable losoxantrone dose of 45 mg m^−2^. With granulocyte colony-stimulating factor support, significant further dose escalation of losoxantrone was achieved. Cardiotoxicity was seen with cumulative dosing. Pharmacokinetics of losoxantrone revealed linear kinetics and triphasic clearance, with significant interpatient variability. No objective responses were seen in this study. Neutropenia was dose-limiting in this combination with or without granulocyte colony-stimulating factor support. The recommended dose for further testing is cyclophosphamide 500 mg m^−2^ followed by losoxantrone 95 mg m^−2^ with granulocyte colony-stimulating factor support.

*British Journal of Cancer* (2002) **86**, 534–539. DOI: 10.1038/sj/bjc/6600123 www.bjcancer.com

© 2002 Cancer Research UK

## 

Losoxantrone (CI-941) is an anthrapyrazole cytotoxic drug that acts through DNA intercalation and topoisomerase II inhibition, resulting in single and double stranded DNA breaks (Fry *et al*, 1985; Showalter *et al*, 1987; Leteurtre *et al*, 1994). Unlike anthracyclines, it does not form reactive free radicals with NADPH, and therefore theoretically should have less propensity for cardiotoxicity through this mechanism (Graham *et al*, 1987). Losoxantrone demonstrated impressive cytotoxic activity in a broad range of tumour cell lines (Leopold *et al*, 1985). Accordingly, losoxantrone was selected for further development with the anticipated potential to replace anthracyclines through a more favourable therapeutic index.

Although anthracycline mediated cardiotoxicity is multifactorial, the major mechanism is likely free-radical-mediated myocyte damage (Doroshow, 1986; Kesavan *et al*, 1996). Although initial experiments in a foetal heart model showed less cardiotoxic effects than doxorubicin (Fagan *et al*, 1984; Graham *et al*, 1987; Fisher and Patterson, 1992), more recent toxicological data in hypertensive rats showed similar histological features and no significant differences in severity of cardiac damage in the two agents (Herman *et al*, 1998). In phase I trials the main dose-limiting toxicity was neutropenia, and a dose of 50 mg m^−2^ q3 weeks bolus infusion was recommended for further testing (Foster *et al*, 1992). Cardiotoxicity was reported to be uncommon. In phase II trials losoxantrone showed promising clinical activity against advanced breast cancer with a 43–63% response rate (Talbot *et al*, 1991; Calvert *et al*, 1994). In these studies, no clinically significant cardiac toxicity was observed. Therefore, further studies to develop losoxantrone were taken to identify active combinations with established agents like paclitaxel (Diab *et al*, 1999) and cyclophosphamide. Cyclophosphamide in combination with doxorubicin is at present the standard treatment for breast cancer. In addition, cyclophosphamide also has an important role in high dose chemotherapy with stem cell transplantation regimens. These were the basis for conducting this phase I trial of cyclophosphamide combined with losoxantrone.

## MATERIALS AND METHODS

### Eligibility criteria

The study opened in October 1992 and closed in September 1996 to patient accrual. Patients 18 years or older with histologically or cytologically proven solid malignancies refractory to standard chemotherapy or for which no standard chemotherapy exists were eligible if they fulfilled the other entry criteria which included: World Health Organization (WHO) performance status 0–2, life expectancy at least 8 weeks, adequate renal (serum creatinine ⩽2 mg dl^−1^), hepatic (bilirubin ⩽1.5 mg dl^−1^), haematologic function (leukocytes ⩾3.0×10^9^ l^−1^, absolute neutrophil count ⩾1.5×10^9^ l^−1^, platelet count ⩾100×10^9^ l^−1^) and cardiac function (defined by baseline LVEF ⩾45% by multiple gated acquisition scan with no decrease ⩾10% in the recent 6 months). Patients with myocardial infarction in the past year, congestive cardiac failure, unstable angina, cardiomyopathy, ventricular arrhythmias on treatment or previous treatment with cumulative doses of doxorubicin more than 300 mg m^−2^ or mitoxantrone more than 125 mg m^−2^ were excluded. Patients should not have received cytotoxic, hormonal therapy or radiotherapy within 3 weeks of study treatment (6 weeks for nitrosoureas and mitomycin C) and should have recovered from drug-induced toxicity from these agents. Other exclusion criteria included severe active medical conditions, pregnancy or lactation. A signed written informed consent was obtained for all patients according to federal and institutional guidelines.

### Treatment plan

Both drugs were administered by bolus intravenous infusions over 10 min each, with cyclophosphamide preceding losoxantrone. Treatment was repeated every 3 weeks until tumour progression or occurrence of unacceptable toxicity (as defined by an absolute fall in the left ventricular ejection fraction from baseline >15% or below 40% or grade 4 nonhaematological toxicity) or at the patient's or physician's discretion.

Cyclophosphamide dose was fixed at 500 mg m^−2^ and losoxantrone dose was escalated from a starting dose of 30 mg m^−2^ through 40, 45 and 50 mg m^−2^. A minimum of three patients were treated at each dose level. Dose-limiting toxicity (DLT) was defined as grade 4 neutropenia lasting more than 5 days or associated with fever; grade 4 thrombocytopenia; or grade 3 or 4 non-haematological toxicity (excluding nausea and vomiting) occurring in the first or second cycle of treatment. If a DLT was observed, two additional patients were treated at that dose-level. Dose escalation proceeded only if less than three out of five patients had dose-limiting toxicity. The dose level at which three or more patients experienced DLT defined the maximum tolerated dose (MTD). No intrapatient dose escalation was allowed, but patients who experienced DLT were allowed further treatment if clinically indicated with dose reduction to the next lower level. Dose delay for recovery from toxicity was also allowed up to 3 weeks from the scheduled day of treatment. Three stages of the study were planned: Initial stage without G-CSF support, second stage with G-CSF support if neutropenia was dose-limiting, and single-agent losoxantrone with G-CSF support in the third stage. G-CSF was administered subcutaneously at 5 μg kg day^−1^ from day 3 of the cycle until neutrophil recovery past nadir to more than 1.5×10^9^ l^−1^.

### Drug administration

Losoxantrone was supplied by DuPont Pharmaceuticals Company (Wilmington, DE, USA) in vials containing 25 mg of lyophilized powder. Each vial was reconstituted with 2.5 ml of sterile water for injection to give a concentration of 10 mg ml^−1^. The calculated total dose for a patient was then further diluted with normal saline to a total volume of 50 ml and administered by infusion over 10 min. All patients received prophylactic antiemetics with serotonin antagonists.

Cyclophosphamide (Cytoxan®) was supplied by the University of Chicago Hospital Pharmacy and administered over 10 min infusion. G-CSF (Neupogen®, Amgen, Thousand Oaks, USA) was supplied as ampules containing 0.6 mg of cytokine in 2 ml of sterile water.

### Evaluation

All patients had baseline history, physical examination, complete blood counts, electrolytes, blood urea nitrogen, creatinine, liver function tests, electrocardiogram, multiple gated acquisition scan (MUGA) and tumour measurements by relevant radiological investigations. Toxicity assessment was done weekly during the study using the National Cancer Institute Common Toxicity Criteria Version 1 for grading. Complete blood counts were performed twice weekly and more frequently during grade 3 or 4 myelosuppression. Blood chemistries were repeated weekly. MUGA scan and tumour assessments were repeated after every two cycles. Response was measured according to the World Health Organization criteria for tumour response (Miller *et al*, 1981).

### Pharmacokinetics and pharmacodynamics

Pharmacokinetic sampling for losoxantrone plasma concentrations were obtained at the following time points during the first cycle of chemotherapy: at baseline and 5, 10, 20, 30, 40, 65 min and at 1.5, 2, 3, 4, 6, 8, 12, 14, 24, 30, 48, 72 and 96 h after the start of the losoxantrone infusion. The blood samples were drawn from an indwelling intravenous cannula from the arm contralateral to that used for drug infusion into plastic centrifuge tubes containing citrate buffer, and centrifuged at 3300 revolutions per minute for 15 min. The resulting plasma supernatant was then separated and stored at −20°C until analysis. Losoxantrone plasma levels were determined using a validated high-performance liquid chromatographic (HPLC) method (Graham *et al*, 1989). Calibration curves were linear within the concentration ranges relevant to this study, with precision and accuracy within 15% and mean extraction efficiency of 99.7±12.7%.

Non-compartmental and compartmental methods were used to derive pharmacokinetic parameters for losoxantrone (Gibaldi and Perrier, 1982). Using the linear trapezoidal method as implemented in PK-IMS Version 2.0 software (DuPont Pharmaceuticals Company), area-under-the plasma concentration-time curve (AUC) extrapolated to infinity, terminal half-life (T1/2), mean residence time (MRT), volume of distribution at steady-state (V_ss_) and systemic clearance (CL) were calculated. Compartmental methods were used to fit losoxantrone concentrations using the software WINNONLIN (Pharsight Corporation, Cary, NC, USA). The pharmacokinetic parameters of losoxantrone from this combination study were compared (however, no statistical comparisons were made between these across study data) with those obtained in two single agent studies (Allen *et al*, 1991; Graham *et al*, 1992), and a study where losoxantrone was administered along with paclitaxel (Diab *et al*, 1999).

Pharmacodynamic analysis explored the relationship between losoxantrone exposure and toxicity or response. A sigmoidal E_max_ model was used to fit the percentage change in neutrophils and the losoxantrone AUC using the equation:


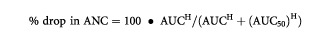


where H is the Hill constant that describes the sigmoidicity of the curve, AUC_50_ is the area-under-the concentration-time curve at half-maximal effect. Percentage fall in LVEF was correlated with cumulative dose per square metre of losoxantrone and actual cumulative dose of losoxantrone using linear regression analysis. The F statistic was tested to determine the significance of correlation.

## RESULTS

Forty-nine patients enrolled in the study, of which 46 were evaluable for toxicity; their characteristics are shown in Table 1

**Table 1:**
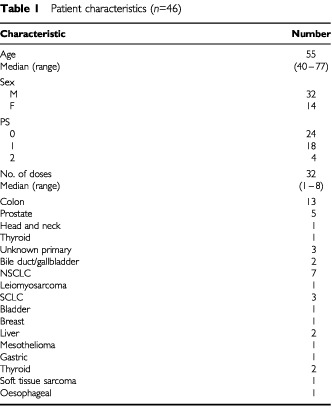
Patient characteristics (*n*=46)

. Two inevaluable patients did not receive treatment after enrolment due to withdrawal of consent and one was ineligible on the day of first treatment due to elevation of bilirubin. The patient population consisted of patients who were fairly heavily pretreated with chemotherapy, of which seven patients had received prior anthracycline therapy; five patients had prior doxorubicin (maximum 240 mg m^−2^), one patient had liposomal doxorubicin 1200 mg m^−2^, and one patient had received 44 mg m^−2^ of mitoxantrone.

Ninety-five cycles of treatment were administered with a median of two cycles per patient over 12 dose levels of losoxantrone, ranging from 30 to 150 mg m^−2^ of losoxantrone ( Table 2

**Table 2:**
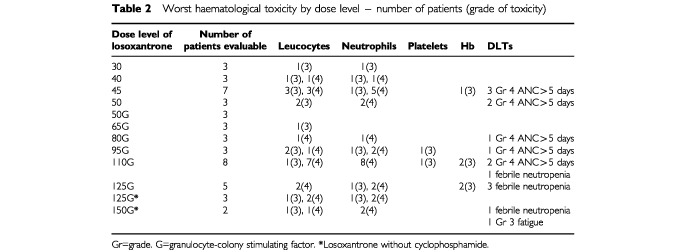
Worst haematological toxicity by dose level – number of patients (grade of toxicity)

). The cumulative dose of losoxantrone ranged from 60 to 570 mg m^−2^, with a median of 225 mg m^−2^.

### Haematological toxicity (Table 2)

Table 2 shows the grade 3 or 4 haematological toxicities at all the dose levels. Dose-limiting neutropenia was observed at doses of 45 and 50 mg m^−2^, with three out of seven patients and two out of three patients experiencing grade 4 neutropenia lasting more than 5 days, respectively. Therefore the MTD was reached at 45 mg m^−2^ of losoxantrone, which is close to its recommended single agent dose. G-CSF ameliorated neutropenia and allowed significant further escalation of losoxantrone dose to 125 mg m^−2^, at which three out of five patients developed febrile neutropenia which defined this dose as the new MTD. The next lower dose cohort treated at 110 mg m^−2^ was expanded to include a total of eight patients, of which three fulfilled the criteria for DLT; two experienced grade 4 neutropenia lasting more than 5 days and one patient had febrile neutropenia. Two further dose levels of losoxantrone without cyclophosphamide, 125 and 150 mg m^−2^ were studied, but the study was terminated before the MTD was reached because of a sponsor decision. However, both patients treated at 150 mg m^−2^ experienced DLT, one with febrile neutropenia and the other with grade 3 fatigue. No DLTs were observed at the next lower dose of 125 mg m^−2^ without cyclosphosphamide. Anaemia was more frequent at the higher dose levels, with two patients developing grade 3 anaemia at 110 and 125 mg m^−2^ respectively. Grade 3 thrombocytopenia occurred in one patient at 95 and 110 mg m^−2^ of losoxantrone, respectively. A dose intensity analysis using data from at least two cycles of treatment showed that 87–91% of the scheduled dose intensity of losoxantrone could be administered at 80 and 95 mg m^−2^ respectively, supporting the tolerability of these doses (Hryniuk, 1996).

### Nonhaematological toxicity

Fatigue, nausea and vomiting grade 2 or less were observed at most dose levels and ameliorated by symptomatic measures. Grade 3 fatigue was experienced by one patient at 150 mg m^−2^ of losoxantrone. A serious but previously unreported toxicity was interstitial lung disease in a 73-year old male patient with metastatic prostatic cancer with no evidence of pulmonary metastases or prior thoracic radiotherapy. He was treated at a dose of 110 mg m^−2^ of losoxantrone (cumulative dose of losoxantrone of 330 mg m^−2^) and developed progressive dyspnea, fever, and dry cough 1 week after the third dose. High resolution CT scan of the lung showed diffuse bilateral upper lobe alveolar infiltrates. Transbronchial biopsy did not reveal any infective etiology. Despite steroids and oxygen treatment, the pulmonary process progressed and he died from respiratory failure 3 months after the last dose of losoxantrone. An autopsy performed showed diffuse alveolar damage.

### Cardiac toxicity

**Figure 1:**
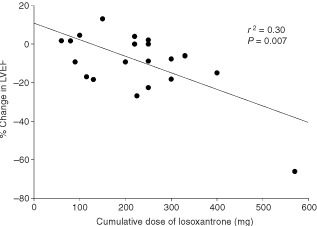
Plot of cumulative dose of losoxantrone against % change in left ventricular ejection fraction. Solid line represents the line of best fit.

Twenty-three patients had paired MUGA assessments, having received at least two cycles of chemotherapy treatment. Figure 1

shows the change of the LVEF as a function of the cumulative dose of losoxantrone. Drop in the left ventricular ejection fraction of more than 10% from the baseline was observed in seven (30%) patients at dose levels 50, 65, 125 and 150 mg m^−2^ of losoxantrone. In one patient, clinically apparent cardiac failure developed 2 months after cessation of losoxantrone treatment. This patient was a 48-year old female with thyroid cancer and no prior anthracycline therapy who received a cumulative dose of 570 mg m^−2^ of losoxantrone, the highest in this study. Transthoracic echocardiogram showed an estimated LVEF of 20% and global hypokinesia, which was a significant drop from the baseline of 59%. Linear regression analysis showed a significant relationship between cumulative dose per m^2^ (*r*=0.62, *P*=0.002) and actual dose (*r*=0.55, *P*=0.007) with percentage change in LVEF. However, this positive relationship was strongly dependent on the data point of the patient with severe cardiac failure at a cumulative dose of 570 mg m^−2^ of losoxantrone. There was no significant relationship between the change in LVEF and the cumulative dose of prior anthracycline therapy (*r*=0.14, *P*=0.2).

### Response

No objective responses were observed in this study. Only one patient had breast cancer that had prior treatment with cyclo phosphamide, doxorubicin and 5-fluorouracil and paclitaxel, and the patient had progressive disease after two treatment cycles on this study.

### Pharmacokinetic and pharmacodynamic analyses

First cycle losoxantrone pharmacokinetics were studied in 31 patients; all dose levels were studied except doses above 110 mg m^−2^. The pharmacokinetic parameters of losoxantrone are listed in Table 3

**Table 3:**
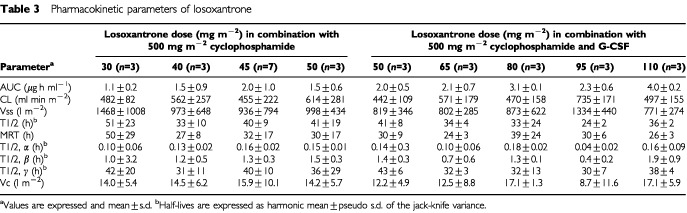
Pharmacokinetic parameters of losoxantrone

**Figure 2:**
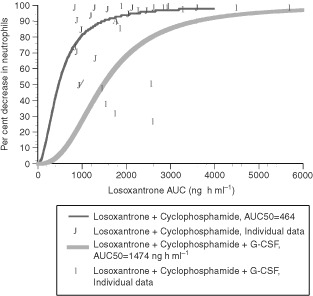
Sigmoidal E_max_ model of % change in neutrophil count at nadir *vs* AUC of losoxantrone.

. A three compartmental model with intravenous infusion input was found to optimally fit data for estimating the half-lives t1/2α, t1/2β and t1/2γ and the central compartment volume of distribution (V_c_). Losoxantrone pharmacokinetics were characterised by a rapid distribution phase into tissue with a t1/2α of ∼6–8 min, t1/2β of ∼1 h and with a prolonged terminal half-life t1/2γ of ∼36 h. Clearance was rapid (average of 526±190 ml min m^−2^ and range of 442 to 735 ml min m^−2^), and steady state volume of distribution was large (average of 989±583 l m^−2^ and range of 771 to 1465 l m^−2^). There was large interpatient variability (% CV ranged from 30 to 60%) in the pharmacokinetics. Clearance appeared to be linear across dose levels, as clearance did not correlate with dose (*r*=0.16, *P*=0.38). A sigmoidal E_max_ model was used to demonstrate the correlation between the per cent change in neutrophil count at nadir in the first cycle and losoxantrone AUC, with and without G-CSF support. As shown ( Figure 2

), there was a shift of the curve to the right with G-CSF support, with approximately tripling in the value of AUC_50_ from 464 to 1474 ng h ml^−1^.

## DISCUSSION

Neutropenia is the dose-limiting toxicity of a 3-weekly short infusion schedule of cyclophosphamide and losoxantrone in this study. We found that with a fixed dose of cyclophosphamide of 500 mg m^−2^, a losoxantrone dose of 45 mg m^−2^ appeared to be tolerable. G-CSF support allowed significant dose escalation of losoxantrone beyond its single-agent recommended phase II dose. MTD was reached at a dose of 125 mg m^−2^ of losoxantrone, with three patients out of a cohort of five patients experiencing DLTs (neutropenic fever) based on toxicities observed in the first two cycles. The next lower dose was therefore expanded to include eight evaluable patients. However, myelosuppression was also significant at this dose level, and one death occurred after the third cycle due to interstitial lung disease possibly attributable temporally to chemotherapy. According to the definition of DLT in this study, three of these toxic events were considered dose-limiting; two patients developing grade 4 neutropenia lasting more than 5 days and one patient with febrile neutropenia. Dose levels of 95 and 80 mg m^−2^ of losoxantrone were tolerable based on limited number of patients treated. On this basis, we recommend a losoxantrone dose of 95 mg m^−2^ when combined with cyclophosphamide at a dose of 500 mg m^−2^ and G-CSF support for further studies in phase II clinical trials. However, because the study was terminated early due to a decision made by the sponsor, we did not have the opportunity to gain more experience with these dose levels. Without cyclophosphamide but with G-CSF, the limited toxicity data on the three patients treated at 125 mg m^−2^ of losoxantrone suggested tolerability at this dose. Therefore, G-CSF allows significant extension of the therapeutic index of losoxantrone.

Uncommon but severe delayed nonhaematological toxicities were encountered, consisting of one patient with overt cardiac failure and another with acute alveolitis respectively. Although it was not definite that losoxantrone was solely responsible for the acute respiratory failure, histological changes found at autopsy was reported as consistent with drug-induced alveolar damage.

Anthracenediones like mitoxantrone were developed as part of the anthracycline analogue programme to identify agents with less cardiotoxicity that maintained antitumour activity (Murdock *et al*, 1979). Mitoxantrone lacked the sugar moiety of anthracyclines, and was selected on the basis of reduced capacity for free radical production (Doroshow and Davis, 1983; Kharasch and Novak, 1983; Nguyen and Gutierrez, 1990). However, subsequent clinical studies reported cardiotoxicity clearly associated with mitoxantrone use (Unvererferth *et al*, 1983; Coleman *et al*, 1984; Mather *et al*, 1987). Anthrapyrazoles were also developed along this same strategy of reduced cardiotoxicity through biochemical structural modification. However, the first developed anthrapyrazole, piroxantrone, was reported to have significant cardiotoxicity in phase II trials in breast cancer (Ingle *et al*, 1994). In a phase II study of an anthrapyrazole piroxantrone conducted by the Southwest Oncology Group in advanced sarcoma, five of 23 patients had reduced cardiac output after treatment, of which one was fatal from congestive cardiac failure (Zalupski *et al*, 1993). Losoxantrone has also been shown to be potentially cardiotoxic in clinical trials, both as a single agent and in combination with paclitaxel. No relationship could be demonstrated between cumulative doses of losoxantrone and change in LVEF in these prior reports. In our study, a correlation between cumulative dose of losoxantrone and reduction in LVEF was found. There are statistical flaws in this analysis, as the trial was not designed to elucidate the effect of losoxantrone dose on changes in LVEF, the sample size was limited, most patients only had two doses of treatment, and that patients would have stopped the drug if a significant drop in LVEF had been detected. Above this, the relationship was also dependent on a single outlier. Therefore, it is too preliminary to conclude that cardiac toxicity from losoxantrone results from cumulative exposure, and to suggest a cumulative dose at which this occurs. However, this data we present would support the existing suspicion that losoxantrone may also be cardiotoxic with cumulative doses. Therefore, anthrapyrazoles have thus far not fulfilled the role of replacing doxorubicin as an active DNA intercalator with minimal or no cardiotoxicity.

The pharmacokinetics of losoxantrone in this study were similar to previous reports. Overall losoxantrone pharmacokinetics was characterized by rapid plasma clearance and a slow rate of elimination suggesting extensive tissue distribution. There was significant interindividual variability in clearance at the various dose levels, as was noted in previous phase I clinical trials of losoxantrone, with or without paclitaxel. No evidence of saturation in kinetics was observed over the range of losoxantrone doses studied. In a single agent phase I study in 23 patients (Graham *et al*, 1992), where losoxantrone dose ranging from 5–55 mg m^−2^ was administered, the plasma clearance averaged 417±194 ml min m^−2^. In another single agent study in 13 patients (Allen *et al*, 1991), where losoxantrone dose ranged from 4–36 mg m^−2^, the plasma clearance averaged 220±89 ml min m^−2^. In the current study where losoxantrone was given with cyclophosphamide, clearance averaged 526±190 ml min m^−2^ and was linear over the dose range of 30 to 110 mg m^−2^. In another phase I study of paclitaxel at 135 mg m^−2^ and losoxantrone at 40 mg m^−2^, 13 patients were studied and the clearance of losoxantrone was 341 ml min m^−2^ (Diab *et al*, 1999). These wide discrepancies in the clearance of losoxantrone are likely due to large interindividual variability. Indeed, this variability in losoxantrone clearance contributed to the unsuccessful utilization of pharmacokinetically guided dose escalation in the dose escalation process in the single agent phase I studies (Graham *et al*, 1992). The reason for the larger interindividual variability of pharmacokinetics is not obvious and deserves further study. Possible contribution from polymorphisms of metabolizing enzymes like the glutathione-S-transferases which are known to exhibit polymorphism and are involved in cyclophosphamide and doxorubicin metabolism, or variability in protein or tissue binding deserves further study.

The per cent reduction in neutrophil nadir could be fit in a sigmoidal E_max_ model against AUC. Using this model, we could show that addition of G-CSF allowed an approximately triple AUC_50_ in a sigmoidal E_max_ model of the per cent reduction of neutrophil nadir against AUC. This effect of G-CSF in improving the therapeutic index of the combination may suggest a possible future role for this combination in high dose chemotherapy protocols. Further investigation of this appears warranted and may revive interest in losoxantrone.

## References

[bib1] AllenSGCummingsJEvansSNicholsonMStewartMECassidyJSoukopMKayeSBSmythJF1991 Phase I study of the anthrapyrazole biantrazole: clinical results and pharmacology Cancer Chemother Pharmacol 28 5558 2040034 10.1007/BF00684957

[bib2] CalvertHSmithIJonesAten Bokkel HuinninkWHedleyDFrancherDAzarinaN1994 Phase II study of losoxantrone in previously treated and untreated patients with advanced breast cancer Proc Am Soc Clin Oncol 13 71

[bib3] ColemanREMaisleyMNKnightRKRubensRD1984 Mitoxantrone in advanced breast cancer – a phase II study with special attention to cardiotoxicity Eur J Cancer Clin Oncol 20 771776 6540179 10.1016/0277-5379(84)90215-3

[bib4] DiabSGBakerSDJoshiABurrisHACobbPWVillalona-CaleroMAEckhardtSGWeissGRRodriguezGIDrenglerRKraynakMHammondLFinizioMVon HoffDDRowinskyEK1999 A phase I and pharmacokinetic study of losoxantrone and paclitaxel in patients with advanced solid tumors Clin Cancer Res 5 299308 10037178

[bib5] DoroshowJH1986 Prevention of doxorubicin-induced killing of MCF-7 human breast cancer cells by oxygen radical scavengers and iron chelating agents Biochem Biophys Res Commun 135 330335 3954778 10.1016/0006-291x(86)90981-2

[bib6] DoroshowJHDavisKJ1983 Comparative cardiac oxygen radical metabolism by anthracycline antibiotics, mitoxantrone, bisantrene, 4'-(9-acridinylamino)-methanesulfon-m-anisidide, and neocarzinostatin Biochem Pharmacol 32 2935 6313012 10.1016/0006-2952(83)90399-4

[bib7] FaganMAHackerMPNewmanRA1984 Cardiotoxic potential of substituted anthra (1,9-cd) pyrazole-6-(2H) ones (anthrapyrazoles) as assessed by the fetal mouse heart organ culture Proc AACR 25 302(abstract 1196)

[bib8] FisherGRPattersonLH1992 Lack of involvement of reactive oxygen in the cytotoxicity of mitoxantrone, CI941 and ametantrone in MCF-7 cells: comparison with doxorubicin Cancer Chemother Pharmacol 30 451458 1394801 10.1007/BF00685596

[bib9] FosterBJNewellDRGrahamMAGumbrellLAJennsKEKayeSBCalvertAH1992 Phase I trial of the anthrapyrazole CI-941: prospective evaluation of a pharmacokinetically guided dose-escalation Eur J Cancer 28 463469 1591064 10.1016/s0959-8049(05)80077-2

[bib10] FryDWBoritzkiTJBessererJAJacksonRC1985 In vitro DNA strand scission and inhibition of nucleic acid synthesis in L1210 leukemia cells by a new class of DNA complexes, the anthra[1,9-cd]pyrazol-6(2H0-ones (anthrapyrazoles) Biochem Pharmacol 34 34993508 2413861 10.1016/0006-2952(85)90724-5

[bib11] GibaldiMPerrierD1982 PharmacokineticsNew York: Marcel Dekker

[bib12] GrahamMANewellDRCalvertAH1989 Determination of the anthrapyrazole anticancer drug CI-941 in plasma and urine by solid-phase extraction and high performance liquid chromatography J Chromatogr Biomed App 491 253261 10.1016/s0378-4347(00)82841-8 2793977

[bib13] GrahamMANewellDRButlerJHoeyB1987 The effect of the anthrapyrazole antitumor agent (CI941) on rat liver microsome and cytochrome P-450 reductase mediated free radical processes. Inhibition of doxorubicin activation in vitro Biochem Pharmacol 36 33453351 2823819 10.1016/0006-2952(87)90309-1

[bib14] GrahamMANewellDRFosterBJGumbrellLAJennsKECalvertAH1992 Clinical pharmacokinetics of the anthrapyrazole CI-941: factors compromising the implementation of a pharmacokinetically guided dose escalation scheme Cancer Res 52 603609 1732048

[bib15] HermanEHHasinoffBBTranKTChadwickDPClarkJrJRFerransVJ1998 Comparison of the chronic toxicity of piroxantrone, losoxantrone and doxorubicin in spontaneously hypertensive rats Toxicology 128 3552 9704904 10.1016/s0300-483x(98)00049-3

[bib16] HryniukWM1996 Dose intensityInPrinciples of Antineoplastic Drug Development and Pharmacology,Schilsky RL, Milano GA, Ratain MJ (eds)pp263279New York: Marcel Dekker Inc

[bib17] IngleJNKurossSAMailliardJALoprinziCLJungSHNelimarkRAKrookJELongHJ1994 Evaluation of piroxantrone in women with metastatic breast cancer and failure on non-anthracycline chemotherapy Cancer 74 17331738 8082075 10.1002/1097-0142(19940915)74:6<1733::aid-cncr2820740615>3.0.co;2-d

[bib18] KesavanSLincoffMYoungJB1996 Anthracycline-induced cardiotoxicity Ann Intern Med 125 4758 8644988 10.7326/0003-4819-125-1-199607010-00008

[bib19] KharaschEDNovakRF1983 Inhibitory effects of anthracenedione antineoplastic agents on hepatic and cardiac lipid peroxidation J Pharmacol Exp Ther 226 500506 6875860

[bib20] LeopoldWRNelsonJMPlowmanJJacksonRC1985 Anthrapyrazoles, a new class of intercalating agents with high-level, broad spectrum activity against murine tumors Cancer Res 45 55325539 4053027

[bib21] LeteurtreFKohlhagenGPaullKDPommierY1994 Topoisomerase II inhibition and cytotoxicity of the anthrapyrazoles DuP937 and Dup941 (losoxantrone) in the National Cancer Institute preclinical antitumor drug discovery screen J Natl Cancer Inst 86 12391244 8040892 10.1093/jnci/86.16.1239

[bib22] MatherFJSimonRMClarkGMvon HoffDD1987 Cardiotoxicity in patients treated with mitoxantrone: Southwest Oncology Group phase II studies Cancer Treat Rep 71 609613 3581099

[bib23] MillerABHoogstratenBStaquetM1981 Reporting results of cancer treatment Cancer 47 207214 7459811 10.1002/1097-0142(19810101)47:1<207::aid-cncr2820470134>3.0.co;2-6

[bib24] MurdockKCChildRGFabioPFAngierRBWallaceREDurrFECitarellaRV1979 Antitumor agents: I. 1, 4-Bis[(aminoalkyl)amino]-9, 10 anthracenediones J Med Chem 22 10241030 490545 10.1021/jm00195a002

[bib25] NguyenBGutierrezPL1990 Mechanism(s) for the metabolism of mitoxantrone: electron spin resonance and electrochemical studies Chem Biol Interact 74 139 2157552 10.1016/0009-2797(90)90064-t

[bib26] ShowalterHDJohnsonJLHoftiezerJMTurnerWRWerbelLMLeopoldWRShillisJLJacksonRCElslagerEF1987 Anthrapyrazole anticancer agents. Synthesis and structure-activity relationships against murine leukemias J Med Chem 30 121131 3806589 10.1021/jm00384a021

[bib27] TalbotDCSmithIMansiJLJudsonICalvertAHAshleySE1991 Anthrapyrazole CI941: a highly active new agent in the treatment of advanced breast cancer J Clin Oncol 9 21412147 1960556 10.1200/JCO.1991.9.12.2141

[bib28] UnvererferthDVUnverferthBJBalcerzakSPBashoreTANeidhartJA1983 Cardiac evaluation of mitoxantrone Cancer Treat Rep 67 343350 6850653

[bib29] ZalupskiMMBenedettiJBalzcerzakSPHutchhinsLFBeltRJHantelAGoodwinJW1993 Phase II trial of piroxantrone for advanced or metastatic soft tissue sarcomas. A Southwest Oncology Group study Invest New Drugs 11 337341 8157477 10.1007/BF00874435

